# A study of dynamic adsorption of propylene and ethylene emitted from the process of coal self-heating

**DOI:** 10.1038/s41598-019-54831-6

**Published:** 2019-12-04

**Authors:** Karolina Wojtacha-Rychter, Adam Smoliński

**Affiliations:** 1 Central Mining Institute, Department of Mining Aerology, Pl. Gwarków 1, 40-166 Katowice, Poland; 2 Central Mining Institute, Pl. Gwarków 1, 40-166 Katowice, Poland

**Keywords:** Environmental sciences, Natural hazards, Solid Earth sciences

## Abstract

The gaseous products emitted in the self-heating process constitute one of the parameters suggested for detecting coal spontaneous combustion in underground mining. The objective of the study is to investigate the changes of ethylene and propylene content in a gaseous mixture which flowed through a fixed bed column filled with bituminous coal of different grain size. The mixtures of fire gases were obtained from laboratory simulated heating of coal at the temperatures of 373 K, 423 K, 473 K and 523 K. Hydrocarbons of various initial concentrations were introduced to the adsorption column at the constant flow rate of 2∙10^−7^ m^3^/s. The experimental findings show that decreasing the adsorbent granulation and gases concentration causes an extended breakthrough and coal bed saturation times. In all the tests, the saturation time was gained faster for ethylene than for propylene. Thus, the content of tested hydrocarbons, which are some of the indicators for assessing the degree of the coal self-heating process, in mine air may change in time as a result of the adsorption phenomenon. It occurs particularly at the early stage of the self-heating process and in places where coal dust has been left.

## Introduction

Endogenous fire hazard is one of the most challenging problems faced in mining activities. It occurs in large masses of coal conducive to the accumulation of heat in places which are not accessible for people; hence, its detection in the early stage of development is very difficult. So far, there have been a number of studies on the process of coal spontaneous combustion^1–8^. The results of these studies showed that the coal self-heating phenomenon initiated by exothermic reaction of coal with oxygen from mine air is the natural cause of mine fires. And the decomposition of active functional groups on coal surface (i.e. hydroxyls –OH, methoxyls –OCH_3_, carboxyls –COOH or carbonyls = CO) during the oxidation process leads to the generation of various gases. Moreover, the coal oxidation rate and the emissions of major gaseous products are affected by physical and chemical properties of coal (i.e. the content of carbon, oxygen, ash, moisture, volatile matter and sulfur) as well as mining factors (coal temperature or ventilation rates). It has been found that during coal self-heating the emission of characteristic fire gases such as carbon monoxide, saturated and unsaturated hydrocarbons, hydrogen and carbon dioxide takes place^9^. The monitoring of the concentration of the oxidation products at various temperatures of coal heating demonstrated that with the increase of coal temperature the concentration of gases increases^10^. Therefore, in recent years, the mine fire detection method has been shifted towards monitoring the concentration of gases in the underground mine environment. Within the framework of the method, various fire indices are calculated to monitor the process of spontaneous combustion and assess the state of its development. The most frequently applied fire indices are the following: Graham’s Ratio, Carbon Dioxide – Oxygen Deficiency Ratio, Willet’s Ratio, Hydrocarbons Ratio, Oxygen Consumption, Carbon/Hydrogen Ratio, Jones and Tricket Ratio, Carbon Monoxide/Carbon Dioxide Ratio^3,11,12^. As can be noticed, the concentrations of the above mentioned fire gases are variables of these indices. The limitation of this method consists in that part of the gaseous products generated from low-temperature oxidation of coal may naturally occur within coal mine atmosphere or can be produced by sources other than the endogenous fire^12,13^. Therefore, the monitoring of atmospheric mine air on a regular basis during normal mining operations constitutes a baseline for daily measurements at the control stations. Also, the daily results of quantitative gas analyses are compared with the reference gas emission profile obtained during laboratory tests of coal heating in the reactor. On the other hand, the concentration of these gases may be lost in mine atmosphere due to dilution caused by ventilation air or adsorption processes, which can cause an incorrect assessment of the fire hazard.

The tendency of gaseous components of mine air to amass on the surface of coal has been previously investigated by means of determining maximum adsorption capacity of coal in relation to single fire gases using the static adsorption technique^14–17^. It has been reported that the contact of gaseous products of the thermal oxidation reaction of coal with an adsorbent such as coal can effectively cause their adsorption in various amounts. The findings presented in research literature show that ethylene, propylene, acetylene and carbon dioxide are sorbed in the largest amount, while the sorption abilities of carbon monoxide and hydrogen are poor^18,19^. In the static method applied in these measurements, the adsorption isotherms described the adsorption effectiveness of the single component from the gas phase and the maximum amounts of gas adsorbed on the degassed coal samples.

Under real conditions, the gases emitted from the sources of coal self-heating constitute a gaseous mixture moving continuously through the coal bed. The determination of the changes of gas concentrations at the time and along the height of the bed is the subject of research on adsorption dynamics^20^. In the studies of dynamic adsorption processes, the adsorbent stream is introduced into a fixed adsorbent layer, usually devoid of the initially adsorbed substance. At the inlet of the sorption column, the bed starts to saturate with the adsorbant to a value which is slightly lower than equilibrium adsorption. With the passage of time, as a result of the continuous flow, the adsorption active layer moves upward the column. The concentration of the gas leaving the column starts to increase and un-adsorbed gas molecules begin to appear in the gaseous mixture at the outlet. The points where the gas at the outlet of the sorption column gains the value of 5 and 95% of the initial concentration are called a breakthrough time (t_b_) and saturation time (t_s_), respectively^21^. The saturation time is the end point of the adsorption where the bed is considered as being exhausted (i.e. more gas cannot be adsorbed).

A few experiments were executed by using the fixed-bed column method. These studies discussed mainly the impact of physical and chemical properties of coals and gas molecules on the amount of sorbed gases from a multi-component mixture^22–25^. In the previous research^25^, only the retention time of ethylene, propylene, ethane and propane on various coal samples at constant gas concentrations and the C_k_/C_o_ ratio equal to 0.6 was determined. The authors found that propylene gained the required value of 0.6 at the most extended time, independently of the coal used as adsorbent. In addition, the influence of coal grain size in a fixed-bed column on hydrocarbon adsorption was dealt with only in terms of sorption capacity^26^.

The objective of the present study is to investigate the dynamic adsorption process of fire gases emitted during the simulation of thermal oxidation of coal. Although carbon monoxide, carbon dioxide and hydrogen as well as unsaturated/saturated hydrocarbons are commonly applied for calculating the values of fire indices and for the assessment of coal self-ignition hazard in Australia, China, U.S. or Poland^3^, our research has been limited to measuring only the changes of propylene and ethylene concentration in the mixture. The influence of the adsorption process on the concentration of these gases in mine air is much stronger than on the concentration of carbon monoxide, hydrogen and saturated hydrocarbons^16,27^. The experiments carried out to determine the sorption capacity of coal in relation to all fire gases indicated that a double bond between carbon atoms in ethylene and propylene molecule may contribute to the high reactivity of these gases. The selection of these unsaturated hydrocarbons was also determined by technical limitations.

The novelty of this study consists in analyzing the influence of two various parameters such as the initial concentration of the gas in the stream moving through the sorption column and the grain size of coal filling the column on the dynamic adsorption of ethylene and propylene coming from a multi-component mixture. Coal grain size is one of the significant factors influencing the amount of sorbed gases. During mining activities, the natural balance of the rock mass is disturbed; this implies the changes of the original distribution of stresses, which causes the fracture of rocks and the formation of new fissures where the fragmented coal can be accumulated. The finer grains created during the crushing of coal cause that the area of contact with the gas from the flowing stream will be larger, and the number of active centers on the internal coal surface and the amount of sorbed gas will increase. However, the impact of coal grain size on the adsorption of a single gas can be different than for gases from the mixture because of competitive adsorption.

The findings of this study could be a scientific reference for future research directions in the field of sorption processes of gases, in particular ethylene and propylene in the mixture.

## Experimental Section

### Materials

Bituminous coal coming from an active coal mine located in the area of the Upper Silesian Coal Basin (USCB), Poland was used as an adsorbent in the sorption column. The proximate, ultimate and petrographic analyses of the tested coal are presented in Tables 1 and 2.

**Table 1:** Physical and chemical results of the coal sample tested.

Parameter	Results	Standard
**Ultimate analysis**		
Carbon, %w/w	77.47	ISO 29541:2010
Hydrogen, %w/w	5.05	ISO 29541:2010
Oxygen, %w/w	7.34	ISO 29541:2010
Nitrogen, %w/w	1.43	ISO 29541:2010
Sulfur, %w/w	0.61	ISO 334:1992
**Proximate analysis**		
Ash, %w/w	5.21	ISO 1171:2010
Moisture, %w/w	2.98	ISO 589:2008
Volatiles, %w/w	34.26	ISO 562:2010
Fixed Carbon, %w/w	57.55	ISO 562:2010
Mineral Matter, %vol.	9	ISO 7404-3:2009

**Table 2:** Petrographic analysis results of the coal sample tested.

Parameter	Results	Standard
Vitrinite, %vol.	68	ISO 7404-3:2009
Liptinite, % vol.	10	ISO 7404-3:2009
Inertinite, %vol.	22	ISO 7404-3:2009

Proximate parameters such as ash, moisture and volatile matter were determined using the LECO TGA 701 or MAC 500 ThermoGravimetric Analyzer, whereas fixed carbon was calculated as the difference of 100% – moisture – ash – volatile matter. The ultimate analysis involved determining the elemental composition of the tested coal i.e. carbon, hydrogen, nitrogen, sulfur and oxygen content. The oxygen content was calculated as the difference of 100% – moisture – ash – carbon – hydrogen, while the remaining parameters were measured with the application of LECO TruSpecCHN and TruSpecS analyzers.

The characteristic of bituminous coal pore structure was performed with the application of Autosorb IQ – Gas Sorption Analyzer (Quantachrome Instruments, Boynton Beach, FL, USA)^28,29^. The nitrogen adsorption isotherms at 77 K (the temperature of liquid nitrogen) were applied in order to determine the porous structure parameters. The specific surface area and pore size distribution were calculated according to the Brunauer, Emmett and Teller (BET) adsorption isotherm equation and the model of Density Functional Theory (DFT), respectively^30,31^.

**Figure 1:**
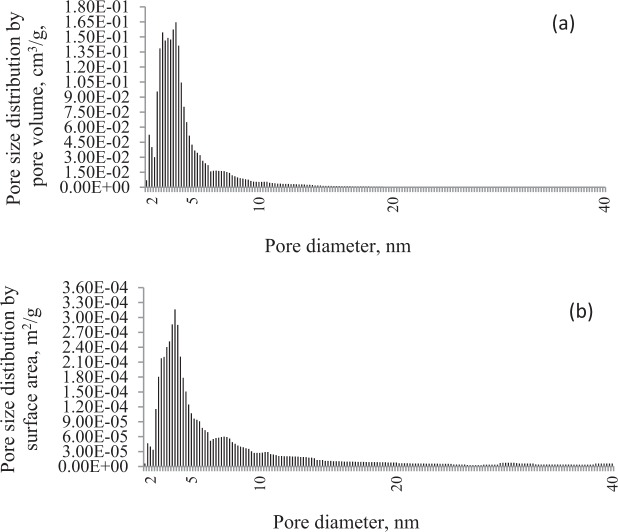
Pore size distribution by (**a**) pore volume and (**b**) surface area of tested coal.

Bituminous coal is characterized by a relatively low specific surface area of 3.88 m2/g, an average pore diameter of 7.71 nm and a total pore volume of 0.007 cm3/g. The analysis of pore size distribution data (Fig. 1) for coal sieved to the particle size range of 0.50–0.70 mm showed a dominant share of mesopores (a unimodal peak between 2 and 5 nm) in the surface area and pore volume. The percentage of micropores surface area and pore volume of coal was only in the range of around 1% and 2.5%, respectively.

**Figure 2:**
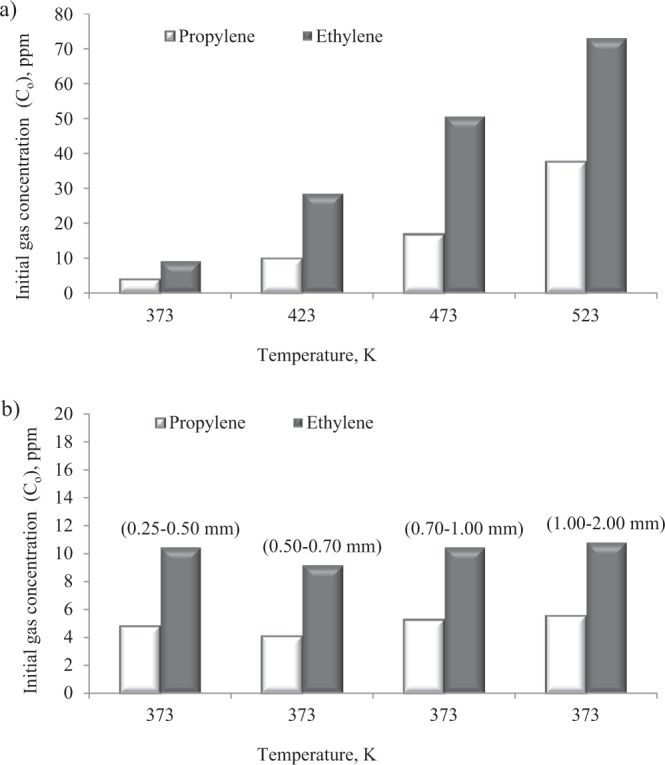
Concentration of ethylene and propylene in a multi-component mixture in the measurement series at (**a)** a different initial gases concentration and (**b)** a different grain size of adsorbent.

Propylene and ethylene coming from the gaseous mixture applied in the laboratory tests as adsorbants were produced as a result of the oxidation of coal in a specially designed apparatus to simulate coal spontaneous combustion under various temperature conditions. The experiment was performed using the method commonly applied for modeling the profile of gaseous products emitted during coal oxidation in the laboratory conditions according to an internal procedure of the Laboratory of Coal Spontaneous Combustion. The procedure has been developed based on many years of experience in the field of fire hazard assessment and national regulation^32^. A sample of coal with particle size below 2 mm and weight of 0.4 kg was placed on a steel mesh located 1 cm above the bottom of an air tight reactor in order to obtain a better distribution of the oxidation agent flow and the sample was heated up to the set temperatures. Then, synthetic air (O_2_–20.5%vol., N_2_ – 79.5%vol.) was injected from the bottom of the reactor. Gaseous products of both the thermal decomposition and oxidation of coal started to pass upwards through the coal bed. At the outlet of the reactor, propylene and ethylene concentrations in the multicomponent gas mixture were measured four times during the course of the experiments. An average concentration value was applied for the calculations. Figure 2(a,b) show the concentration of each of the unsaturated hydrocarbons in the mixtures. It was found that the analyzed samples reached the values of the ratio of ethylene to propylene concentration in the range of 2–3. A similar relationship between the gases can be found in the work^33^. In the measurement series with a different particle size of coal in a fixed–bed (Fig. 2(b)), the mean value of ethylene and propylene concentration in the gas stream was equal to 10.24 (±0.72) ppm and 4.95 (±0.63) ppm, respectively. As we can notice in Fig. 2(a), the hydrocarbons concentration increases with the temperature of coal heating in the reactor. In the dynamic adsorption tests, the concentration of ethylene and propylene in the mixtures increased from 9.19 ppm to 73.18 ppm and from 4.12 ppm to 37.90 ppm, respectively, with the rise of coal temperature from 373 K to 523 K.

### Experimental setup and procedure

The dynamic adsorption studies of ethylene and propylene in a fixed bed column of bituminous coal were carried out with the application of a laboratory scale installation of the Laboratory of Coal Spontaneous Combustion at the Department of Mining Aerology. The experimental test was performed based on the combination of two methods, i.e. a method that is widely adopted in our laboratory to study coal oxidation and coal spontaneous combustion products, and the fixed-bed adsorption method by means of which the change of gas concentration versus time is analyzed^8,34–37^.

**Figure 3:**
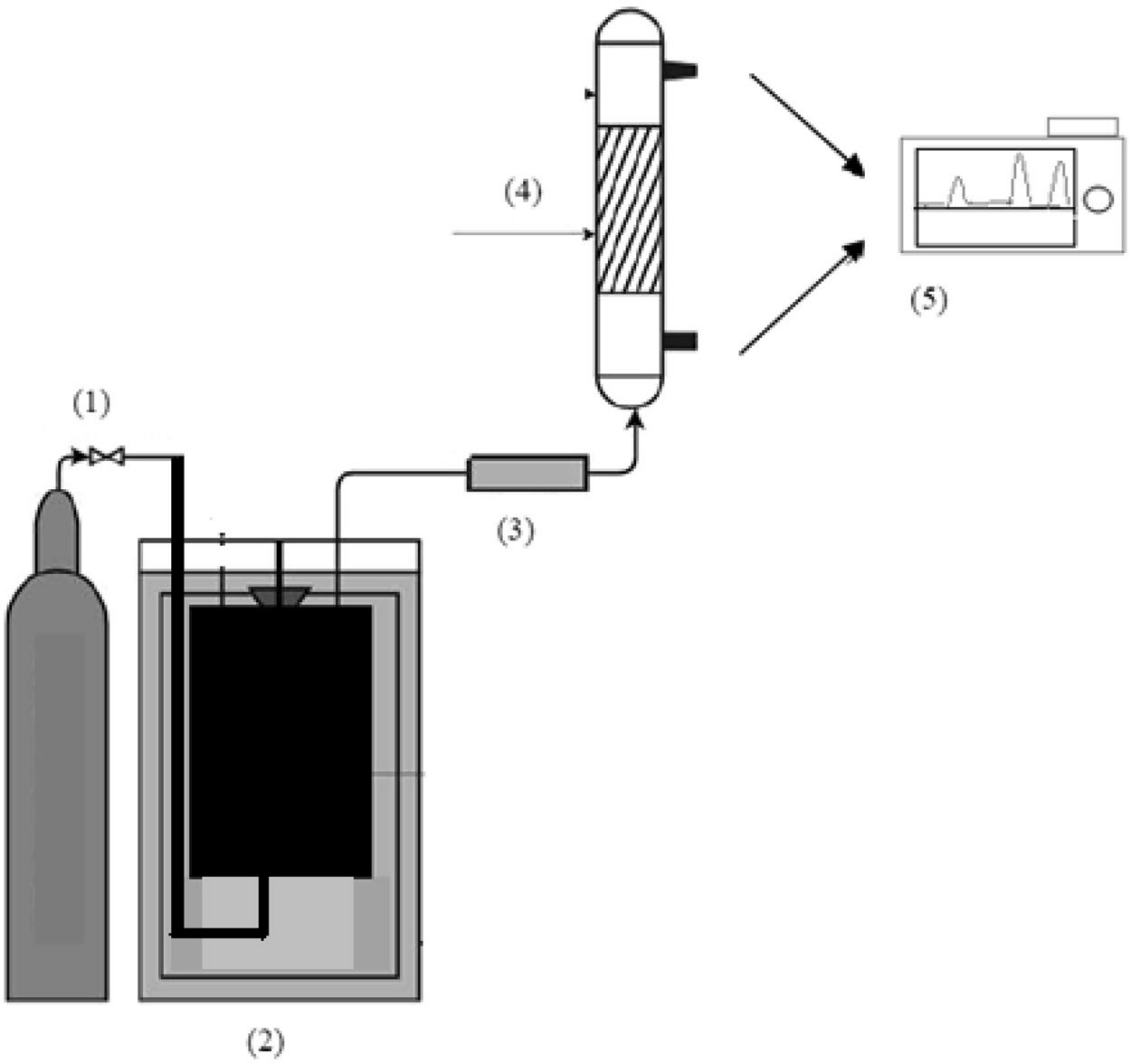
Schematic diagram of the laboratory–scale installation for measuring dynamic sorption of gases.

The test stand presented in Fig. 3 consists of the following elements: a supply system of an oxidation agent with flow controller (1), a fixed bed reactor with a volume of 0.7 L heated with a resistance furnace (2), a flow meter (3), a sorption column with a cross section of 4.9 cm2 (4) and a gas chromatograph (5).

The dynamic sorption experiment consisted in a continuous upward flow of the oxidation reaction products of coal through a borosilicate glass column packed with a crushed coal sample. At the outlet of the sorption column, the gaseous samples were collected manually in a Tedlar bag at regular time intervals. The hydrocarbons concentrations were measured every 5 min (for the first three samples), every 15 min (for the next six samples) and every 30 min (for the last three samples) for the maximum time of 195 min. According to an internal procedure, the synthetic air is injected into the reactor filled with a crushed coal sample with the flow rate of 6∙10^−7^ m^3^/s. A low flow rate of air does not provide enough oxygen for the oxidation reaction while too high a rate causes the dissipation of heat, and, at the same time, it leads to the cooling of the system. This set flow rate of synthetic air determined the final flow rate of gaseous mixture at the outlet of the reactor. All sorption measurements were conducted at the set flow rate of hydrocarbons mixture of 2.2∙10^−7^ m^3^/s controlled by means of ALICAT Scientific MS–Series Mass Flow Meters.

A gas chromatography technique was applied to determine the gas concentration at the outlet and the inlet of the sorption column. The chromatograph (model Hewlett-Packard–HP 6890 GC System, Agilent Technologies Inc.) consists of a Flame Ionization Detector (FID) and two independent output channels equipped with a chromatography packed column of Activated Alumina F1. The reason why the FID detector-type gas chromatograph was applied is the fact that it is very sensitive to most hydrocarbons^38^. The samples were injected into the chromatograph in the amount of 20 ml each. Pure helium was used as the carrier gas with a constant flow mode of 30 ml/min. The column temperature was programmed to the range of 323–473 K. The total amount of hydrocarbons adsorbed by a gram of the adsorbent (q) was calculated according to the following Eq. (1) ^39^

1 $$q=\frac{\frac{Q}{1000}\cdot {\int }_{0}^{t}({C}_{o}-{C}_{t})\cdot dt}{m}$$

where Q (ml/min) is a volumetric flow rate, C_o_ (mg/l) is the initial gas concentration, C_t_, (mg/l) is a gas concentration at any given time t, m is the mass of coal in the column and t (min) denotes time.

To investigate the influence of gas concentrations on breakthrough and saturation times, a coal sample was heated to the following set process temperatures of 373 K, 423 K, 473 K and 523 K. The adsorption column was packed with 200 g of bituminous coal with the grain size range of 0.50–0.70 mm. In order to determine the impact of the degree of fragmentation on the gas adsorption process in coal structure, the sample packed in a fixed-bed column was ground and separated by means of sieves into four mesh sizes, 0.25–0.50 mm, 0.50–0.70 mm, 0.70–1.00 mm and 1.00–2.00 mm. In the measurement series with the application of different grain size of coal, a mixture with a similar share of hydrocarbons obtained as a result of the oxidation of coal at the temperature of 373 K was applied.

## Results and Discussion

### The influence of hydrocarbons concentration on their adsorption

**Figure 4:**
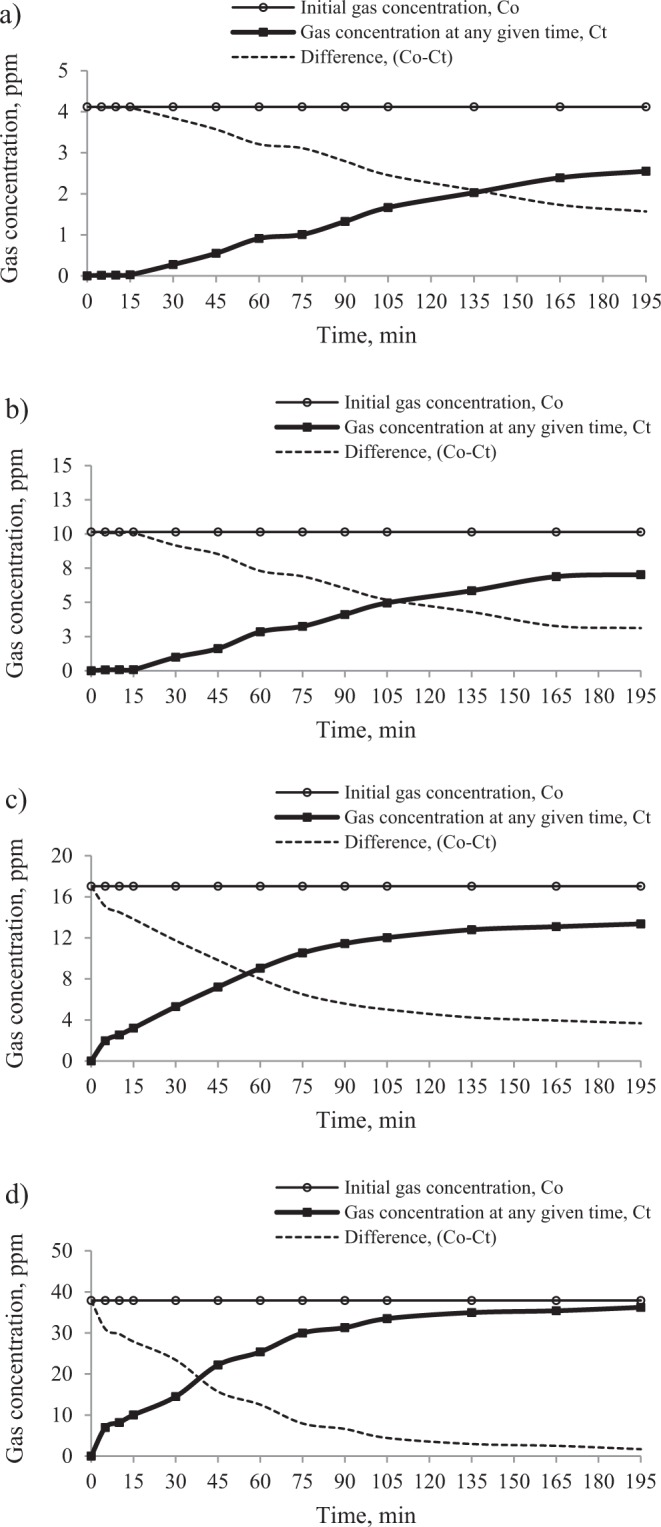
The effect of initial gas concentration on the changes of propylene concentration at the outlet of the sorption column. The temperature of coal heating in the reactor: (**a)** 373 K, (**b)** 423 K, (**c)** 473 K and (**d)** 523 K.

**Figure 5:**
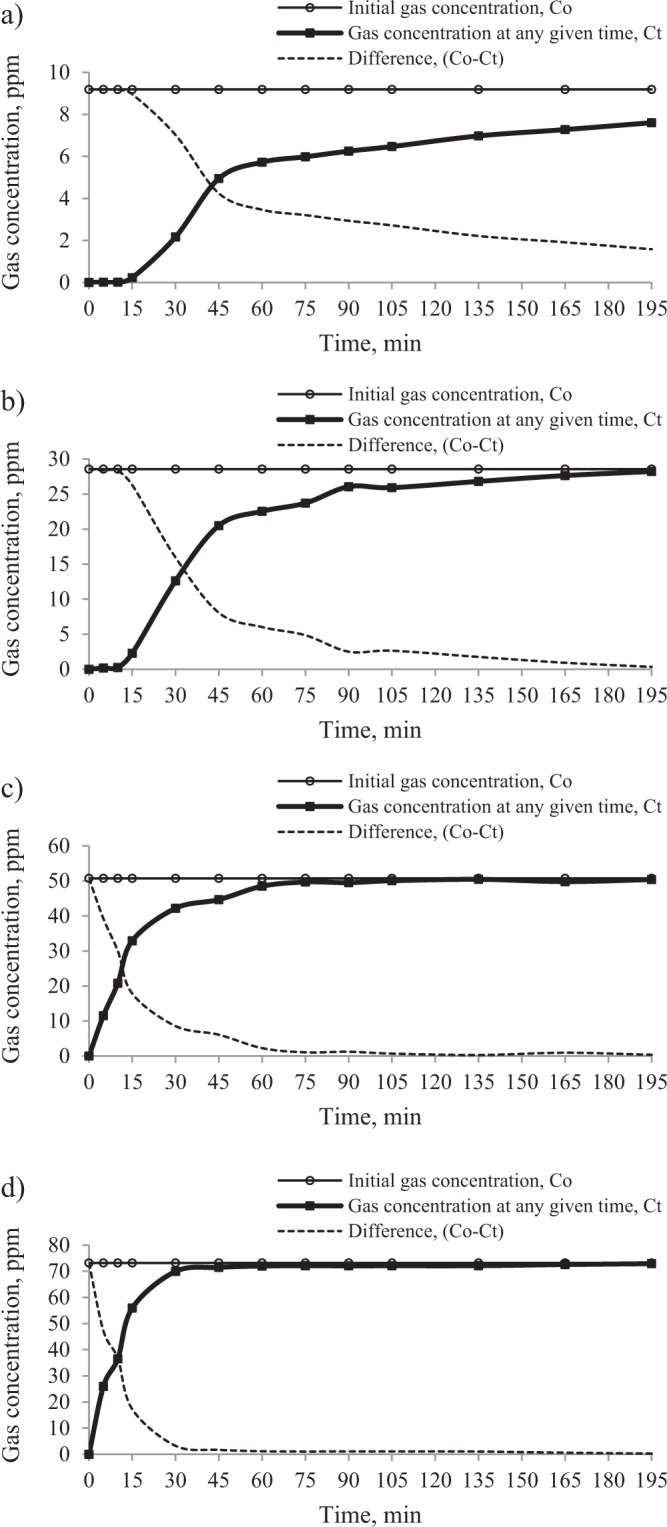
The effect of initial gas concentration on the changes of ethylene concentration at the outlet of the sorption column. The temperature of coal heating in the reactor: (**a)** 373 K, (**b)** 423 K, (**c)** 473 K and (**d)** 523 K.

Figures 4(a–d) and 5(a–d) illustrate the curves plot, respectively for the propylene and ethylene concentrations at the inlet and at the outlet (after moving through the sorption column) as well as their difference as a function of time.

**Figure 6:**
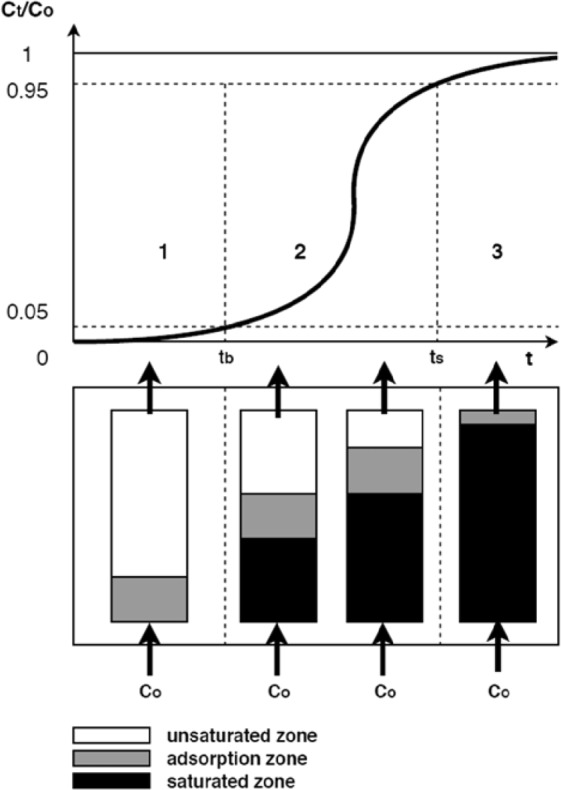
The movement of the adsorption zone in the fixed bed column.

In theory, the dynamic adsorption process, illustrated in Fig. 6, consists of three stages^36,40^. In the first stage (1), the adsorption zone is formed at the beginning of the column and the gas concentration in the outlet stream is close to zero. In the curve, this stage is shown as a straight line parallel or inclined at a slight angle in relation to the X axis. As time goes, the active adsorption zone starts to move towards the end of the column (2). The ratio of the gas concentration at any given time and the initial concentration (C_t_/C_o_) gradually increases from 0.05 to 0.95. In the third stage (3), the column is completely saturated and the adsorption process no longer occurs. The gas concentration in the outlet stream is equal or close to the initial concentration (C_t_≈C_o_). As can be noticed in Fig. 6, the curve resembles the graph of a logistic function (a characteristic “S” shape)^41^.

In the studies, the curves representing the changes of gas concentration at the outlet show an S-shape for lower initial concentrations, (Figs. 4(a,b) and 5(a,b)) and stage (1) is clearly seen, while for higher concentrations range (Figs. 4(c,d) and 5(c,d)) these curves resemble the shape of a logarithmic function and stage (3) is clearly seen. The shape of the curves for higher concentration proves that the breakthrough point was reached much more rapidly (below 5 minutes); it is observed only in stage (2) and (3). The results display that the slope of the ethylene curves in relation to the X axis (stage (2)) is steeper than for propylene, indicating a shorter travel time of the gas. A similar trend was observed in a previous work^25^. As can be seen in Figs. 4(a–d) and 5(a–d), the moment of achieving the adsorption equilibrium (stage (3)) is strongly influenced by the initial gas concentration and the type of the gas. It may be noted that saturation time (95% of the initial concentration) was obtained faster for ethylene than propylene and for higher values of the initial gas concentrations. Interestingly, after ethylene reached equilibrium, the sorption process for propylene was still observed; this implies that the equilibrium amount of adsorbed ethylene may constitute only a small part of the adsorption capacity of coal.

Tables 3 and 4 present the tests results of the adsorption of ethylene and propylene on coal with the application of the fixed-bed technique. A breakthrough time (t_b_), a middle time (t_0.5_) and a saturation time (t_s_) are the times when the gas concentration at the outlet of the sorption column equaled 5%, 50% and 95% of the initial concentration, respectively^36,40,42^.

**Table 3:** Measurement results for the adsorption of propylene in different process conditions.

Grain size of coal d (mm)	Initial gas concentration, C_o_ (ppm)	Adsorption capacity, q (mg/g)	Breakthrough time, t_b_ (min)	Middle time, t_0.5_ (min)	Saturation time, t_s_ (min)
0.5–0.7	4.12	6.46∙10^−5^	30	145	>195
0.5–0.7	10.15	1.42∙10^−4^	24	129	>195
0.5–0.7	17.03	1.62∙10^−4^	<5	66	>195
0.5–0.7	37.90	2.27∙10^−4^	<5	41	142
0.25–0.5	4.83	1.02∙10^−4^	55	>195	>195
0.5–0.7	4.12	6.46∙10^−5^	30	145	>195
0.7–1.0	5.29	7.45∙10^−5^	25	117	>195
1.0–2.0	5.57	6.16∙10^−5^	18	91	>195

**Table 4:** Measurement results for the adsorption of ethylene in different process conditions.

Grain size of coal d (mm)	Initial gas concentration, C_o_ (ppm)	Adsorption capacity, q (mg/g)	Breakthrough time, t_b_ (min)	Middle time, t_0.5_ (min)	Saturation time, t_s,_(min)
0.5–0.7	9.19	5.72∙10^−5^	10	51	>195
0.5–0.7	28.57	1.02∙10^−4^	8	33	130
0.5–0.7	50.75	7.07∙10^−5^	<5	9	80
0.5–0.7	73.18	6.46∙10^−5^	<5	7	50
0.25–0.5	10.46	1.05∙10^−4^	35	129	>195
0.5–0.7	9.19	5.72∙10^−5^	10	51	>195
0.7–1.0	10.46	5.05∙10^−5^	8	39	>195
1.0–2.0	10.83	3.33∙10^−5^	7	30	118

The results in Table 3 show that propylene did not reach the 95% of the initial concentration at the outlet of the sorption column in the established time range (0–195 min), except for the highest concentration, for which the saturation time (t_s_) was 142 min. The values of the ratio of propylene concentration at the time of 195 min (C_t_ = 195) and at the inlet of the sorption column (C_o_) were 0.62 (for the initial concentration of 4.12 ppm), 0.69 (for the initial concentration of 10.15 ppm) and 0.78 (for the initial concentration of 17.03 ppm). In the case of ethylene, see Table 4, the saturation time (t_s_) of the adsorbent bed was reached for higher concentrations, while for the lowest concentration of 9.19 ppm the ratio of C_195_/C_o_ equaled 0.83. On the basis of the above results, it can be also observed that with the increase of gases concentration in the mixture, the values of middle (t_0.5_) times decrease. For propylene, the middle time changed from 145 min to 41 min, while for ethylene it decreased from 51 min to 7 min. At higher propylene concentrations (17.03 ppm and 37.90 ppm) and higher ethylene concentrations (50.75 ppm and 73.18 ppm), the breakthrough point of coal bed occurred at the time below 5 minutes. Therefore, it was not possible to demonstrate the accurate breakthrough time for the higher concentrations. The values of the ratio of propylene concentration at the time of 5 min (C_t_ = 5) and at the inlet of the sorption column (C_o_) were 0.12 (for the initial concentration of 17.03 ppm) and 0.18 (for the initial concentration of 37.90 ppm), while for ethylene the values were 0.23 (for the initial concentration of 50.75 ppm) and 0.35 (for the initial concentration of 73.18 ppm). Based on the above results, it can be also assumed that increasing the gas concentration makes that the breakthrough point will be reached more quickly. The higher concentration of hydrocarbons in the mixtures caused that a much larger number of active centers on the surface area is occupied, which led to much faster exhaustion of adsorption sites inside the coal structure and a quicker achievement of the breakthrough point. Increasing propylene and ethylene concentration in the mixtures affects the increase of loading rate of these gases and the driving force for mass transfer^43,44^. The driving force is defined as the difference between the gas concentration in streamed gas and the gas concentration near the surface of the adsorbent^45^. Therefore, at the beginning of the process, the value of the driving force is equal to the initial gas concentration (C_o_). Hence, it can be concluded that the higher gas concentration at the inlet of the sorption column, the faster the gas moves through the coal bed.

It can be noticed from Tables 3 and 4 that the breakthrough time of the fixed-bed was 3–4 times shorter for ethylene than for propylene. The longer time in the case of propylene means slower transportation of propylene through coal due to the lower gas concentration in the mixture (Fig. 2(a)). The results given in Fig. 2(a) demonstrate that the concentration of propylene was 2–3 times lower than ethylene. Moreover, the sorption process of the other gas components in the mixture with high concentration e.g. ethane, propane or carbon dioxide may also cause the swelling of the coal matrix and decrease the permeability of the transport channels for propylene^46–48^. The results of the tests displayed in Tables 3 and 4 indicate that with the increase of gas concentrations at the inlet of the sorption column, the amount of propylene adsorbed generally increases, while the amount of ethylene adsorbed changed irregularly. The presented research findings show that within the set time range, the sorption capacity of ethylene was lower than propylene. And the value of the difference increased with increasing the concentration of propylene. It can be assumed that the increasing propylene concentration in the mixture caused an increase in the driving force and a decrease in the number of active places available for ethylene adsorption because propylene has a better sorption ability as compared to ethylene. The differences between hydrocarbons properties were discussed in more detail in the works^22,25,33^. The higher reactivity of propylene can be connected with higher critical temperature and more stable secondary carbocation which is formed from propylene during the addition reaction^16,49^.

Tests carried out with various gas concentrations led to the conclusion that at the initial stage of the self-heating of coal, when the concentration of hydrocarbons is low, the saturation of coal will run slowly resulting in an extended breakthrough time of coal bed, particularly for propylene. It implies that the gas emission profile obtained during the reference coal heating under laboratory conditions will be different from the one obtained under real conditions, which may affect the correctness of coal self-heating assessment and the gas content in mine air will be underestimated. Under laboratory conditions, the influence of the adsorption process is not taken into consideration. Gases emitted from the reactor as a source of coal self-heating are directly collected and submitted to the chromatographic analysis. Meanwhile, in underground mine conditions, the gaseous products migrate through the porous coal bed and are subject to adsorption in different amounts, particularly when the distance between the sampling point and the source of coal self-heating is long. This is due to the fact that as the distance increases, the number of active sites available for the adsorption of gases and gas contact time with the coal surface increase.

### Effect of grain size of coal on hydrocarbons adsorption

**Figure 7:**
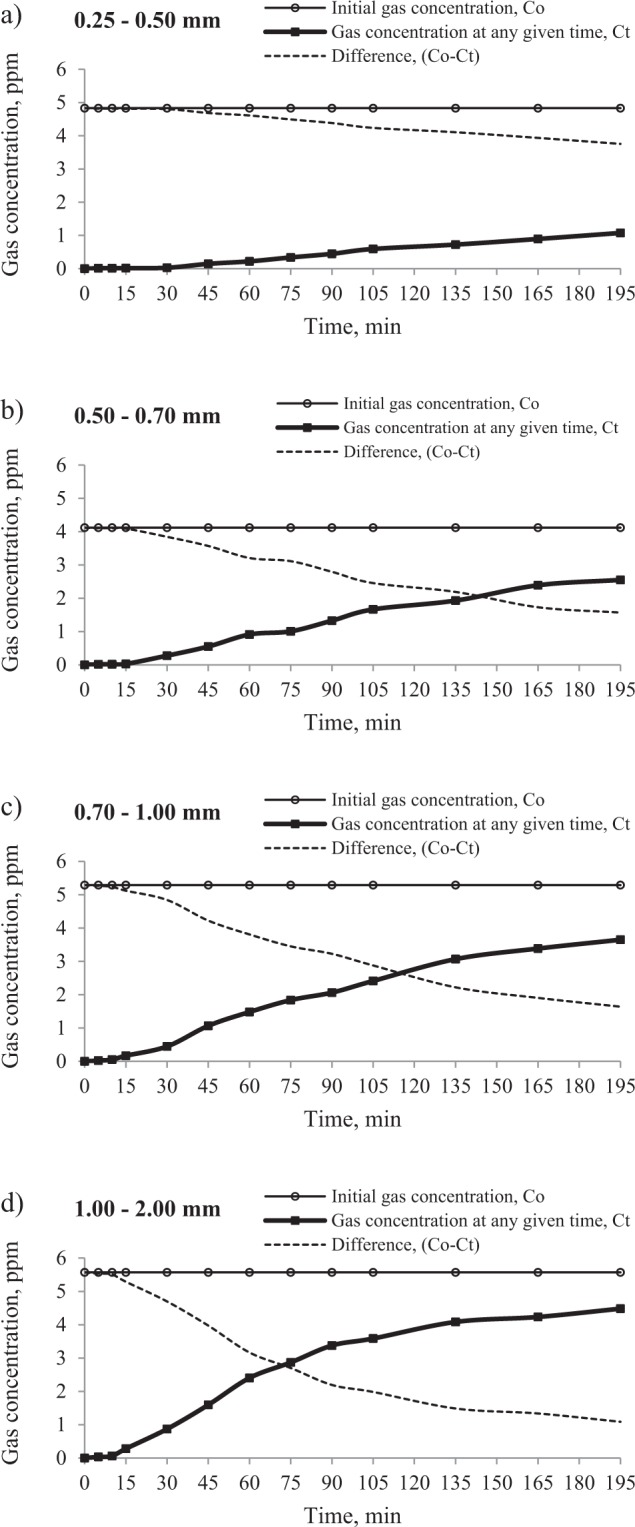
The effect of grain size of adsorbent on the changes of propylene concentration in the mixture moving through the sorption column filled with a coal sample of grain size: (**a)** 0.25–0.50 mm, (**b)** 0.50–0.70 mm, (**c)** 0.70–1.00 mm and (**d)** 1.00–2.00 mm.

**Figure 8:**
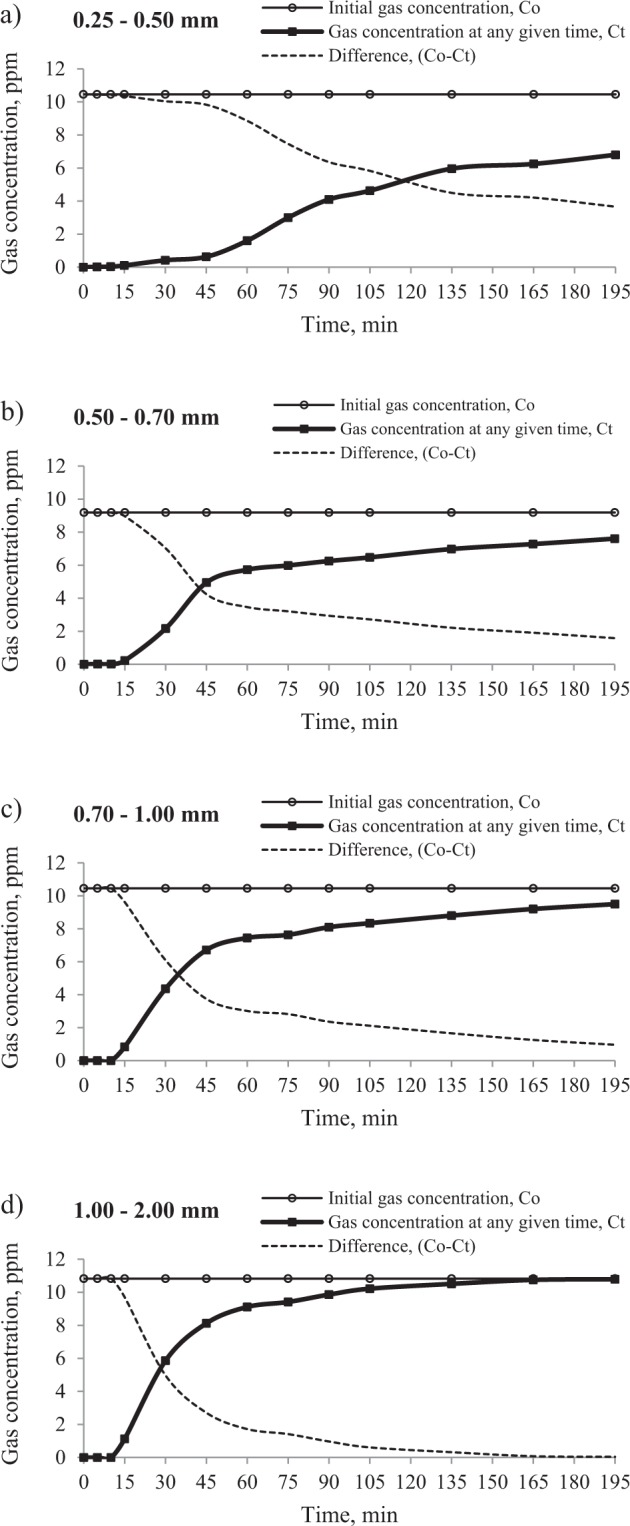
The effect of grain size of adsorbent on the changes of ethylene concentration in the mixture moving through the sorption column filled with a coal sample of grain size: (**a)** 0.25–0.50 mm, (**b)** 0.50–0.70 mm, (**c)** 0.70–1.00 mm and (**d)** 1.00–2.00 mm.

Figures 7(a–d) and 8(a–d) reveal that the curves of propylene and ethylene are clearly shifted to the right and the steepness of the curves decreases with the decreasing grain size of the adsorbent due to the fact that the adsorption process is a surface phenomenon^50,51^. Decreasing the particle size enlarges the surface area and consequently the formation time of the adsorption zone increases. Additionally, during the fragmentation process, the number of accessible pores can increase as the closed pores connect.

The angle of the inclination of propylene curves representing the changes of gas concentration at the outlet is much smaller than that of ethylene. It can result from both the propylene lower concentration and its larger diameter. Therefore, it can be assumed that with the varied character of pore structure in coal, the flow of propylene may be impeded, particularly in narrow flow channels. It was found that for ethylene and the grain size of 1.00–2.00 mm, the equilibrium state was reached; see Fig. 7(d), while in the entire time range propylene did not achieve the equilibrium state.

As indicated in Tables 3 and 4, in the finer grain size range of 0.25–0.50 mm, the breakpoint of bed by ethylene and propylene was observed latest. Decreasing the particle size of coal prolonged the breakthrough time (t_b_) as well as the middle time (t_0.5_) but increased the amount of adsorbed hydrocarbons. For example, the change of the particle size range of coal from 0.25–0.50 mm to 0.50–0.70 mm and from 0.50–0.70 mm to 0.70–1.00 mm resulted in the reduction of breakthrough time for propylene by about 25 min and 5 min, respectively, while for particle size of 0.70–1.00 mm and 1.00–2.00 mm the breakpoint difference was shorter by 7 min. The delayed breakthrough and the slower saturation of the bed in the case of a finer grain size of coal are caused by the larger specific surface area in comparison with the coarse grains. It is commonly known that the reduction of particle size of coal leads to the increase of both the surface area, and, simultaneously of the number of active centers responsible for polar interactions of coal with gas molecules, which consequently increases the amount of adsorbed gases^51–53^. The adsorption zone of mass transfer is an area where the sorption process takes place. At the beginning of the process, the adsorption zone is formed in the first layer of the bed at the inlet of the column. As time goes, the gas fills up all the available sites and next the active zone starts to move through the bed. The breakthrough point occurs when the concentration of the gas leaving the column starts to increase as the un-adsorbed gas molecules begin to appear in the gaseous mixture at the outlet. Therefore, the higher sorption capacity of coal with finer particle size increases the formation time of the adsorption zone of mass transfer, and, consequently leads to slower retention of the gas from the column. Besides, the crushing process may facilitate the connection of the closed or isolated pores and enable the access to new active centers^50^. The results of this study show that the breakthrough time (t_b_) of propylene decreased from 55 to 18 min as the grain size increased from 0.25 to 2.0 mm for a mean inlet propylene concentration of about 5 ppm. In the case of ethylene, the breakpoint decreased from 35 to 7 min as the grain size was increased from 0.25 to 2.0 mm for a mean inlet ethylene concentration of about 10 ppm. Therefore, the research findings indicate that the retention of ethylene is 2 to 4 times quicker than that of propylene. This phenomenon can be again assigned to a nearly twofold lower concentration of propylene in gaseous mixtures (Fig. 2(b)), and consequently a slower driving force.

The highest values of sorption capacity of coal were observed for ethylene and propylene at the 0.25–0.50 mm grain size, which was caused by a larger number of available adsorption sites. For the finest grain size, the sorption capacities of both gases were comparable. For propylene, the sorption capacity was 1.02∙10^–4^ mg/g at the gas concentration of 4.83 ppm, while the amount of ethylene taken up by the coal amounted to 1.05∙10^−4^ mg/g at the gas concentration of 10.46 ppm.

The research findings lead to the conclusion that the flow of hydrocarbons from the mixture emitted during the self-heating of coal may have caused the decrease in the concentration of propylene and ethylene at the sampling point underground, especially when the gas moves through the places where coal dust is accumulated.

## Conclusion

The experiments performed and presented in the study provide evidence that the continuous flow of propylene and ethylene from the mixture produced in the reactor causes the adsorption of both the gases on the surface area of coal. For the examined concentration range of propylene and ethylene, the sorption capacity of coal in relation to these gases was small, at a comparable level of 10^−4^–10^−5^ mg/g. In this research, the highest adsorption capacity for propylene was 2.27∙10^−4^ mg/g, whereas for ethylene it was 1.05∙10^−4^ mg/g.

Both the gas concentration and grain size of coal were found to be very influential variables. Based on the results, it can be concluded that at the initial stage of the low-temperature coal oxidation process (low concentrations of the gases), the decrease in propylene and ethylene concentration under real condition can be significant due to the long time of the coal bed saturation with these gases. Comparing the middle times, it was observed that an increase in the inlet concentration from 4.12 to 37.90 ppm (for propylene) and from 9.19 to 73.18 ppm (for ethylene) resulted in a considerable reduction of the residence time of gases in column by about 72% and 86%, respectively. The breakthrough times for propylene and ethylene were prolonged by about 67% and 80%, respectively, with the decrease of particle size of coal from 1.0–2.0 mm to 0.25–0.50 mm. The increase in the gas concentration with the increase of coal heating will increase the amount of adsorbed hydrocarbons while reducing its coal bed saturation time; in such a case, the effect of the sorption of hydrocarbons on the coal can be completely unnoticeable at the monitoring stations in coal mines. It was observed that coal showed different saturation times in respect to propylene and ethylene. Propylene characterized of about 2–4 fold slower breakthrough of coal bed and an extended saturation time compared to ethylene. At the highest gas concentrations, the time at which coal was no longer able to sorb more propylene was reached 92 min later than for ethylene. Thus, referring to the experimental results, it can be said that in coal mines, the change in ethylene concentration caused by its adsorption on coal surface can be unnoticeable as the breakthrough and saturation times for this gas are relatively shorter than for propylene.

The future extension of this research should be channeled towards the dynamic adsorption of hydrocarbons on coal at a different bed height and flow rate of gaseous mixture. It should also focus on how the other gases present in the mixture can affect the retention of ethylene and propylene in order to better recognize the behavior of the gases under real conditions.

## References

[1] Avila C, Wu T, Lester E (2014). Estimating the spontaneous combustion potential of coals using thermogravimetric analysis. Energ. Fuel..

[2] Li J, Fu P, Zhu Q, Mao Y, Yang C (2018). A lab-scale experiment on low-temperature coal oxidation in context of underground coal fires. Appl. Therm. Eng..

[3] Xu T (2017). Heat effect of the oxygen-containing functional groups in coal during spontaneous combustion processes. Adv. Powder Technol..

[4] Singh RVK (2013). Spontaneous heating and fire in coal mines. Procedia Eng..

[5] Taraba B, Michalec Z (2011). Effect of longwall face advance rate on spontaneous heating process in the gob area – CFD modeling. Fuel.

[6] Onifade M, Genc B (2018). Spontaneous combustion of coals and coal-shales. Int. J. Min. Sci. Technol..

[7] Arisoy A, Beamish B (2015). Mutual effects of pyrite and moisture on coal self-heating rates and reaction rate data for pyrite oxidation. Fuel.

[8] Yuan L, Smith AC (2013). Experimental study on CO and CO_2_ emissions from spontaneous heating of coals at varying temperatures and O_2_ concentrations. J. Loss Prevent. Proc..

[9] Yu-guo W, Jian-ming W (2011). Experimental study on significant gases of coal spontaneous combustion by temperature programmed (TP). Procedia Eng..

[10] Adamus A, Šancer J, Guřanová P, Zubiček V (2011). An investigation of the factors associated with interpretation of mine atmosphere for spontaneous combustion in coal mines. Fuel Process. Technol..

[11] Liang Y, Zhang J, Wang L, Luo H, Ren T (2019). Forecasting spontaneous combustion of coal in underground coal mines by index gases: A review. J. Loss Prevent. Proc..

[12] Levi, T., Beamish, B., Brown, R., Theiler, J. & Pope, J. Early detection of spontaneous combustion using laboratory gas evolution tests. *Proceedings of the 24th International Mining Congress of Turkey*, IMCET **2015**, 206–211(2015).

[13] Zhu H, Chang M, Wang H (2017). Study on primal CO gas generation and emission of coal seam. Int. J. Min. Sci. Technol..

[14] Baran P, Cygankiewicz J, Krzyżanowski A, Zarębska K (2013). Sorption of saturated and unsaturated hydrocarbons on selected coal sample from the Pniówek mine. Geology Geophysics & Environ..

[15] Cygankiewicz J, Dudzińska A, Żyła M (2009). The relation between the size of bituminous coal particles and the sorption of carbon monoxide. Miner. Resour. Manage..

[16] Cygankiewicz J, Dudzińska A, Żyła M (2012). Examination of sorption and desorption of hydrogen on several samples of Polish hard coals. Adsorption.

[17] Sripada P, Khan MM, Ramasamy S, Trivedi J, Gupta R (2018). Influence of coal properties on the CO_2_ adsorption capacity of coal gasification residues. Energy Sci. Eng..

[18] Dudzińska A (2019). Analysis of sorption and desorption of unsaturated hydrocarbons: Ethylene, propylene and acetylene on hard coals. Fuel.

[19] Dudzińska A (2017). Sorption properties of hard coals with regard to gases present in the mine atmosphere. J. Earth Sci..

[20] Ahmad AA, Hameed BH (2010). Fixed-bed adsorption of reactive azo dye onto granular activated carbon prepared from waste. J. Hazard. Mater..

[21] Marzbali MH, Esmaieli M (2017). Fixed bed adsorption of tetracycline on a mesoporous activated carbon: Experimental study and neuro-fuzzy modeling. J. Appl. Res. Technol..

[22] Wojtacha-Rychter K, Smoliński A (2017). Sorption characteristic of coal as regards of gas mixtures emitted in the process of the self-heating of coal. E3S Web of Conferences.

[23] Wojtacha-Rychter K, Smoliński A (2018). Multi-component gas mixture transport through porous structure of coal. Fuel.

[24] Wojtacha-Rychter K, Smoliński A (2018). The interaction between coal and multi-component gas mixtures in the process of coal heating at various temperatures: An experimental study. Fuel.

[25] Wojtacha-Rychter K, Smoliński A (2019). Selective adsorption of ethane, ethylene, propane and propylene in flammable gas mixtures on different coal samples and implications for fire hazard assessments. Int. J. Coal Geol..

[26] Wojtacha‐Rychter K, Howaniec N, Smoliński A (2019). The effect of coal grain size on the sorption of hydrocarbons from gas mixtures. Int. J. Energy Res..

[27] Dudzińska A, Cygankiewicz J (2015). Analysis of adsorption tests of gases emitted in the coal self-heating process. Fuel Process. Technol..

[28] Smoliński A, Howaniec N (2017). Analysis of porous structure parameters of biomass chars versus bituminous coal and lignite carbonized at high pressure and temperature – A chemometric study. Energies.

[29] Howaniec N (2016). The effects of pressure on coal chars porous structure development. Fuel.

[30] Rouquerol, F., Rouquerol, J., Sing, K. S. W., Llewellyn, P. & Maurin, G. *Adsorption by powders and porous solids, principles, methodology and applications*. Second ed., Academic Press (2014).

[31] Ambroz F, Macdonald TJ, Martis V, Parkin IP (2018). Evaluation of the BET theory for the characterization of meso and microporous MOFs. Small Methods.

[32] State Mining Authority. Assessment of the state of work safety, mining rescue and safety in connection with mining and geological activities in 2018. Katowice 2019.

[33] Wojtacha-Rychter K, Smoliński A (2018). Research on a gas index reflecting the sorption process on carbon materials in coal mines. Sustainability.

[34] Ravi VR, Ting X (2018). Ren. Study of the susceptibility of coal for spontaneous combustion using adiabatic oxidation method. Chem. Eng. Trans..

[35] Mohalik NK, Lester E, Lowndes IS (2016). Review of experimental methods to determine spontaneous combustion susceptibility of coal – Indian context. Int. J. Min. Reclamat. Environ..

[36] Patel H (2019). Fixed-bed column adsorption study: a comprehensive review. Appl. Wat. Sci..

[37] Salman JM, Njoku VO, Hameed BH (2011). Batch and fixed-bed adsorption of 2,4-dichlorophenoxyacetic acid onto oil palm frond activated carbon. Chem. Eng. Trans..

[38] de Castro Vasconcellos, P., da Rocha, G. O., Caramão, E. B., Machado, M. E., & Krause, L. C. Chromatographic Techniques for Organic Analytes. *In Comprehensive Analytical Chemistry*. Elsevier (2015).

[39] Unuabonah EI, El-Khaiary MI, Olu-Owolabi BI, Adebowale KO (2012). Predicting the dynamics and performance of a polymer–clay based composite in a fixed bed system for the removal of lead (II) ion. Chem. Eng. Res. Des..

[40] Barros, M. A. S. D., Arroyo, A. P. & Silva, A. E. General aspects of aqueous sorption process in fixed beds. In *Mass transfer - advances in sustainable energy and environment oriented numerical modeling*. InTech, Rijeka, 361–386 (2013).

[41] Kucharavy D, De Guio R (2015). Application of logistic growth curve. Procedia Eng..

[42] Chatterjee A, Schiewer S (2011). Biosorption of cadmium(II) ions by citrus peels in a packed bed column: effect of process parameters and comparison of different breakthrough curve models. CLEAN - Soil, Air, Water.

[43] Nouri H, Ouederni A (2013). Modeling of the dynamics adsorption of phenol from an aqueous solution on activated carbon produced from olive stones. Int. J. Chem. Eng. Appl..

[44] Chowdhury ZZ, Zain SM, Rashid AK, Rafique RF, Khalid. K (2012). Breakthrough curve analysis for column dynamics sorption of Mn(II) ions from wastewater by using Mangostana garcinia Peel-Based Granular-Activated. Carbon. J. Chem..

[45] Akpomie KG, Dawodu FA, Adebowale KO (2015). Mechanism on the sorption of heavy metals from binary-solution by a low cost montmorillonite and its desorption potential. Alex. Eng. J..

[46] Nie X, Chen J, Cao Y, Gong D, Deng H (2018). Analysis of coal swelling deformation caused by carbon dioxide adsorption based on X-Ray computed tomography. Geofluids.

[47] Siriwardane HJ, Gondle RK, Smith DH (2009). Shrinkage and swelling of coal induced by desorption and sorption of fluids: Theoretical model and interpretation of a field project. Int. J. Coal Geol..

[48] Vandamme M, Brochard L, Lecampion B, Coussy O (2010). Adsorption and strain: The CO_2_-induced swelling of coal. J. Mech. Phys. Solids.

[49] McMurry, J. E. *Organic chemistry with biological applications*. Second ed., Cengage Learning (2014).

[50] Zou W, Zhao L, Zhu L (2012). Adsorption of uranium (VI) by grapefruit peel in a fixed-bed column: experiments and prediction of breakthrough curves. J. Radioanal. Nucl. Chem..

[51] Dudzińska A, Howaniec N, Smoliński A (2017). Effect of coal grain size on sorption capacity with respect to propylene and acetylene. Energies.

[52] Dudzińska A, Howaniec N, Smoliński A (2015). Experimental study on sorption and desorption of propylene on Polish hard coals. Energ. Fuel..

[53] Lutyński M, González MA (2016). Characteristics of carbon dioxide sorption in coal and gas shale – The effect of particle size. J. Nat. Gas. Sci. Eng..

